# Case report: Ewing sarcoma with EWSR–ERG fusion elevates procalcitonin extremely in the long term without infection

**DOI:** 10.3389/fonc.2022.1047738

**Published:** 2023-01-13

**Authors:** Ying Chen, Tao Qin, Yan Chen, Ming Gao

**Affiliations:** ^1^Sun Yat-sen Memorial Hospital, Sun Yat-sen University, Guangzhou, China; ^2^Department of Medical Oncology, Sun Yat-sen Memorial Hospital, Sun Yat-sen University, Guangzhou, China; ^3^Department of Hematology, The Eighth Affiliated Hospital of Sun Yat-sen University, Shenzhen, China; ^4^Department of Radiology, Sun Yat-sen Memorial Hospital, Sun Yat-sen University, Guangzhou, China

**Keywords:** Ewing sarcoma, EWSR, procalcitonin, infection, case report, inflammatory marker

## Abstract

**Background:**

Ewing sarcoma (ES) represents a rare, aggressive bone and soft-tissue cancer. Unlike breast, liver, pancreatic, and prostate cancers, Ewing sarcoma has had no representative tumor marker until now. The use of procalcitonin (PCT) as a tumor marker is also rarely reported. PCT is a clinically recognized and widely used inflammatory marker in recent years. In rare cases, PCT may also be falsely positive due to non-infectious factors. In the few previously reported papers regarding the correlation between tumors and PCT, we learned that abnormalities of PCT level can also be impacted by individual cancers.

**Case presentation:**

Here, we first reported a case of Ewing sarcoma with markedly elevated PCT without infection and carried out some literature review. The patient was a middle-aged man with extraskeletal Ewing sarcoma whose lesion was located in the distal abdominal ileum. He had a sudden and unprovoked onset of high fever during chemotherapy before surgery. After multiple examinations, the patient’s blood routine, C-reactive protein, blood culture, and CT examination showed no signs of infection, and even the culture from the end of the central venous catheter showed no pathogen growth. Only PCT increased dramatically to more than 200 ng/ml. PCT remained at this level for several months until a single abdominal lumpectomy was performed before it dropped to near-normal levels.

**Conclusion:**

In our report, PCT is significantly elevated in Ewing sarcoma in the absence of infection. Not only that, but we particularly highlighted the precipitous drop in PCT following tumor resection.

## 1 Introduction

Ewing sarcoma was first defined as an independent type of tumor in the early 20th century; it is also known as the Ewing sarcoma family of tumors (ESFT), which mainly includes classical Ewing sarcoma of the bone (ESB), extraskeletal Ewing sarcoma (EES), and primitive neuroectodermal tumor (PNET). It most often begins in the long bone diaphysis, pelvis, and scapula, although it infrequently occurs in soft tissue. All of them share the common cellular histology and genetic mechanism characterized by small round cells expressing high levels of CD99 and Ewing sarcoma breakpoint region 1 protein (EWSR1) gene disruption. This provides a reliable basis for clinical diagnosis of Ewing sarcoma (1–3). Ewing sarcoma is a highly malignant tumor, most common in people aged 10 to 25 years, with a rare occurrence in older adults (4, 5). Patients with this cancer usually manifest pain, swelling, mass, unexpected tiredness, fever with no known cause, unintentional weight loss, or even anemia only (6). With the improvement of multimodality therapy, the overall survival (OS) has increased from approximately 70% a few years ago to approximately 80% in the past 2 years, although 5-year OS was 30% for patients with metastatic disease and less than 10% for patients who relapsed within 2 years of diagnosis (7–9).

Procalcitonin (PCT) is a prohormone consisting of 116 amino acids with a molecular weight of approximately 13 kDa. Under normal circumstances, PCT is secreted by thyroid C cells and then degraded by enzymes into calcitonin, with very little of it entering the peripheral blood (10, 11), while the PCT increases significantly after bacterial infection since multiple tissues can all express the PCT during sepsis (12). However, it is worth noting that elevated PCT levels can also be caused by non-infectious factors in the minority (13). There have been reports of it, and they include but are not limited to extensive surgery (14), pancreatitis (15, 16), end-stage renal disease (17), newborn (within 48 h of birth) (18), autoimmune diseases, and several neoplastic diseases (small-cell lung cancer, medullary thyroid C cell tumor, and cirrhosis combined with hepatic carcinoma) (19–22).

To the best of our knowledge, high serum levels of PCT have not been reported in Ewing sarcoma especially in the circumstances of no meaningful evidence of bacterial infection until now. Here, we present an adult male patient with extraskeletal Ewing sarcoma who had extremely high PCT levels (>200 ng/ml). Another mystery, in this case, is that the markedly elevated PCT value, which lasted for nearly 100 days, plunged precipitously after the resection of the lesion. This expands the clinical value of PCT in Ewing sarcoma. At the same time, in the absence of bacterial infection, Ewing sarcoma should be included in the differential diagnosis of elevated PCT. The patient has provided permission to publish information about his case, and the identity of the patient has been protected. This study was reported in agreement with the principles of the CAse REports (CARE) guidelines (23).

## 2 Case presentation

**Figure 1:**
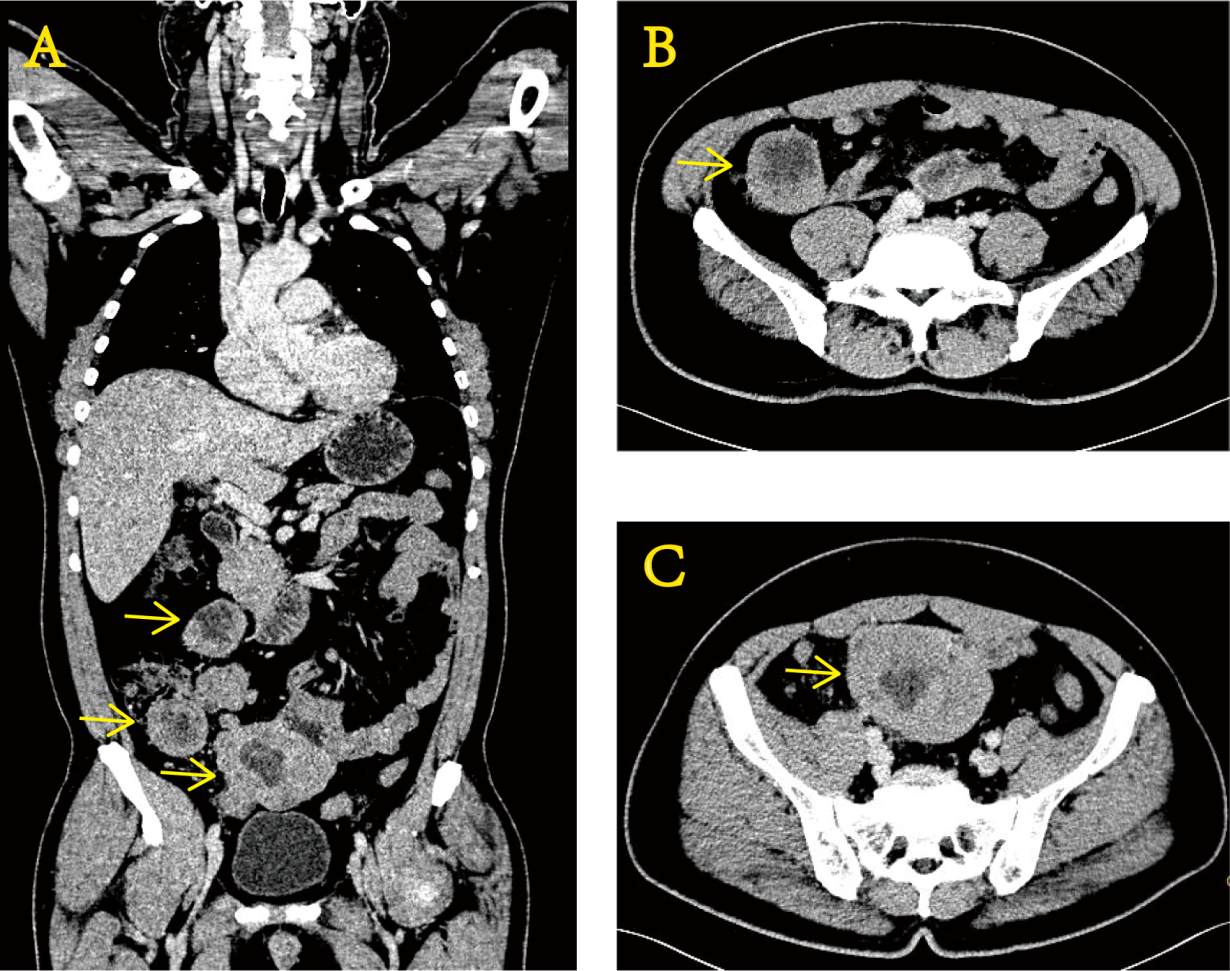
Enhanced CT images of the abdomen at the time of initial diagnosis show uneven thickening of the terminal ileum wall. Coronal section **(A)** and transverse section **(B, C)**.

In November 2020, a 51-year-old man was examined at a local hospital for moderate anemia (hemoglobin level of 80 g/L) after a routine physical examination. Abdominal computed tomography (CT) findings showed uneven thickening of the terminal ileum (**Figure 1**). Positron emission tomography–computed tomography (PET/CT) showed a local soft tissue mass at the distal ileum with abnormally elevated glucose metabolism, which was considered a malignant lesion of the small intestine with multiple lymph node metastases in the abdominal cavity. Then, a puncture aspiration biopsy of the abdominal neoplasm was performed on 3 December 2020. Immunohistochemistry (IHC) showed CD99 (+), vimentin (+), S-100 (+), CD117 (+), partial non-specific enolase (NSE) weakness (+), TdT (−), TTF-1 (−), CgA (−), and WT-1 (−). Fluorescence *in situ* hybridization (FISH) molecular assay revealed the presence of EWSR1 gene disruption. With immunohistochemical and molecular results combined, Ewing sarcoma was confirmed (4).

For further treatment, the patient presented to the oncology department of our hospital on 9 December 2020. We did a preliminary evaluation of the patient. On physical exam, he was lucid, generally in good condition, and without fever, and his vital signs were within the normal range. Cardiopulmonary and abdominal examination showed no obvious abnormalities. Laboratory results showed white blood cells 6.4 × 10^9^/L, red blood cells 4.73 × 10^12^/L, hemoglobin 93 g/L, and platelets 301 × 10^9^/L. Liver and kidney function was normal. Tumor markers were normal except for carbohydrate antigen 125 (45.1 U/ml), which was slightly elevated. Since PCT is not routinely required, we did not initially test for it. Based on the surgeon’s consultation, we decided to administer chemotherapy first, closely review it during chemotherapy, and then change the treatment measures according to the evolution of the disease. According to the National Comprehensive Cancer Network (NCCN) guidelines, we selected the standard first-line treatment includes chemotherapy with a five-drug regimen of vincristine, Adriamycin, and cyclophosphamide, and alternating ifosfamide and etoposide (VAC/IE) for the patient with this tumor and began on the second day of hospitalization. On 9 February 2021, the patient had a sudden high fever without obvious causes outside the hospital. The auxiliary examination at the local hospital showed no abnormalities in blood routine, C-reactive protein (CRP), and blood culture. Also, the CT showed no signs of abscess or infection. Only PCT levels were shockingly over 200 ng/ml. To determine the cause of the fever, peripherally inserted central catheter (PICC) tubes were removed for culture. As in the blood culture, no pathogenic bacteria were found. After 8 days of treatment with ceftazidime and levofloxacin at the local hospital, the patient’s temperature returned to normal and without any abnormal clinical symptoms or signs. However, the PCT levels were still over 200 ng/ml. Over the next 3 months or so, several imaging reviews were performed, and there was no finding of infection. Meanwhile, the efficacy assessment was stable disease (SD). During the same period, the PCT level was repeatedly reviewed also, with no exception, always over 200 ng/ml. In addition, other infection indicators were normal as usual, including CRP less than 5 mg/L each time, serum amyloid A (SAA) less than 4.8 mg/L each time, and white blood cells approximately 5 × 10^9^/L each time. The patient no longer had a fever or any discomfort.

**Figure 2:**
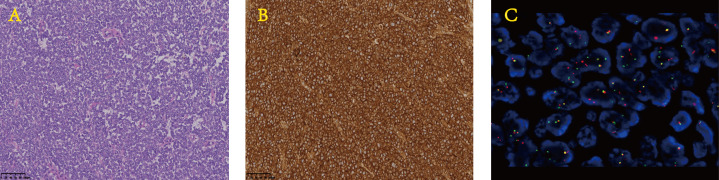
Pathological image of surgical specimens. **(A)** H&E. **(B)** CD99. **(C)** Detection of EWSR1 gene disruption by FISH. The probe type: Vysis LSI EWSR1 Probe (LOT 455960). Result of the patient’s EWSR1 gene disruption test: positive (count 200 cells, approximately 90% show separation signal). FISH, fluorescence *in situ* hybridization.

**Figure 3:**
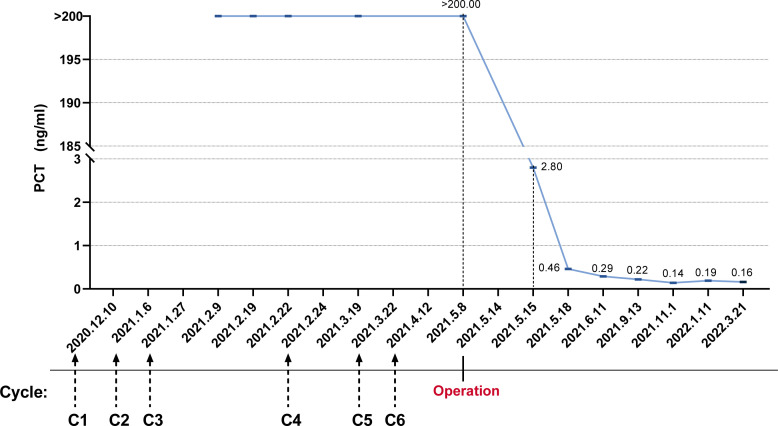
The trend of procalcitonin levels was from abnormally elevated initially to nearly normal after surgical resection.

On 6 May 2021, the patient returned to our hospital again for efficacy evaluation. Up to now, he has completed six cycles of chemotherapy of the five-drug regimen of VAC/IE. The enhanced magnetic resonance imaging (MRI) of his abdomen still indicates SD. Combined with the consultation opinions of gastrointestinal surgery and repeated film review, it was found that there is no engulfment of vessels of each lesion including lymph nodes, so it was considered that the abdominal mass could be removed. For the record, the PCT level at this time is still greater than 200 ng/ml. Other routine laboratory findings include white blood cells 5.29 × 10^9^/L, red blood cells 3.87 × 10^9^/L, hemoglobin 100 g/L, and platelets 243 × 10^9^/L. Serum creatinine and transaminase were normal, and serum albumin and serum calcium levels were normal. The patient had no fever or discomfort. After the exclusion of surgery-related contraindications, the patient underwent abdominal mass resection smoothly on 14 May 2021. Ewing sarcoma was diagnosed again by postoperative pathology. Immunohistochemistry showed CD99 (+), S-100 partial (+), CK minority (+), P53 partial weak (+), Syn minority (+), Ki67 approximately 60% (+), and CgA (−). Molecular assay (FISH) showed positive EWSR1 gene disruption (**Figure 2**). Unexpectedly, laboratory examination revealed 2.80 ng/mL of PCT on the first postoperative day, and there were no other special abnormalities except for a certain degree of increase in the white blood cells (17.35 × 10^9^/L), which was considered a normal postoperative change. Stunningly high levels of PCT, which lasted nearly 100 days in the absence of infection, dropped almost a hundredfold after one operation (**Figure 3**).

**Figure 4:**
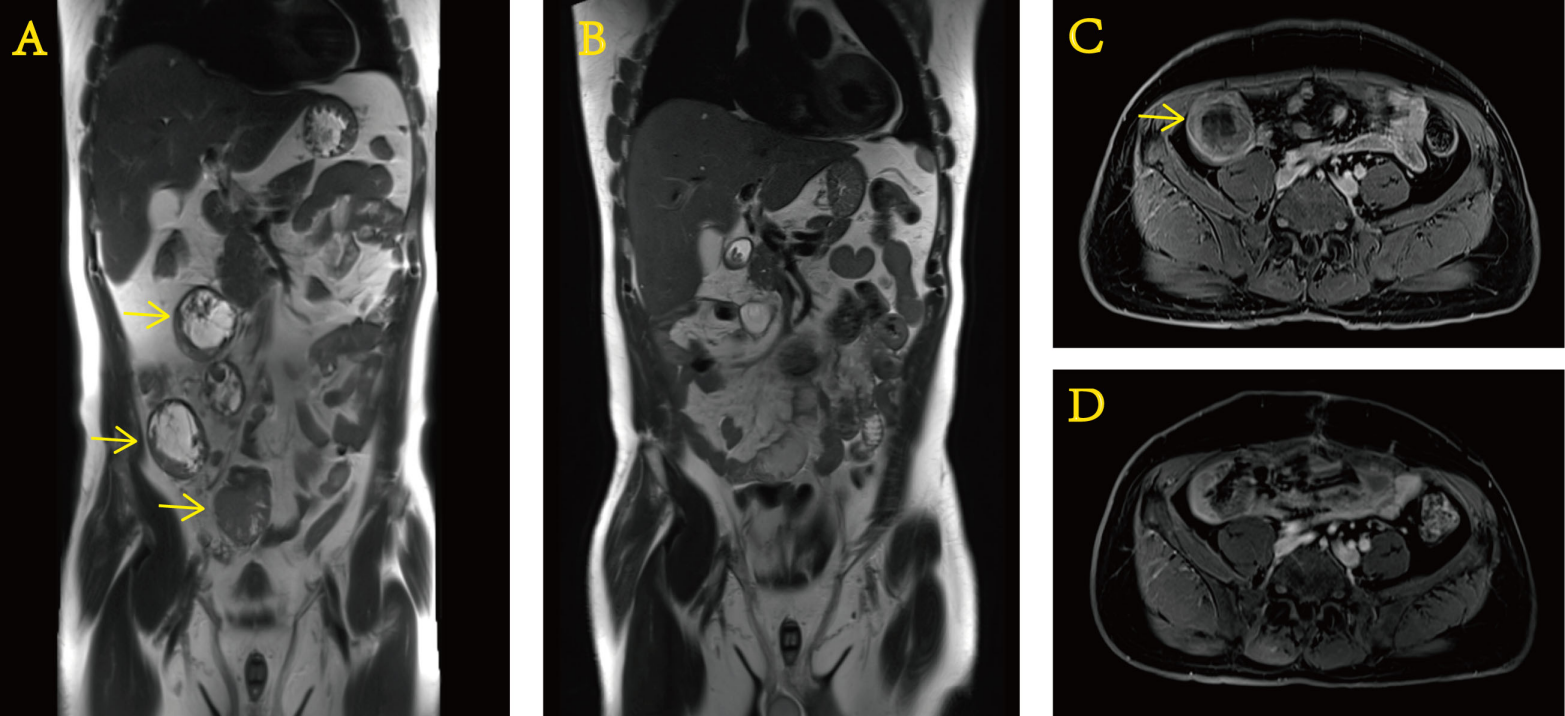
Preoperative and postoperative contrast-enhanced MRI of the abdomen. Preoperative **(A)** coronal and **(C)** transverse sections. Postoperative **(B)** coronal and **(D)** transverse sections.

After approximately 0.5 months, we received the gene test report of the patient’s tumor specimen and peripheral blood based on the detection method of 520-gene panel next-generation sequencing (NGS). The results showed EWSR–ERG gene rearrangement (fusion). After this operation (**Figure 4**), the patient regularly returned to the hospital for postoperative adjuvant chemotherapy with a two-drug regimen consisting of albumin-bound paclitaxel and gemcitabine. The efficacy assessment during each review was the status of no evidence of disease (NED). In addition, it cannot be ignored that the PCT level did not rise anymore. It never went above 0.5 ng/ml again. Until now, in March 2022, the patient still presents to our hospital for re-examination to monitor the change of the disease.

Finally, we reiterate that we have the patient’s oral and written consent, which could confirm that the investigators obtained informed consent to publish information and images from the participant.

## 3 Discussion

At this point, we might marvel at the rarity of this case. Epidemiologically, the patient with Ewing sarcoma was a 51-year-old middle-aged Asian man, not a child or teenager of European descent (24–26). In terms of location, the tumor is of soft tissue origin rather than bone, not to mention trunk or long bone (1, 4). Based on cytogenetic and molecular genetic information, it is EWSR1–ERG that drives epigenetic reprogramming, rather than EWSR1–FLI1, the most common fusion protein, which accounts for 85% (4, 25).

The intimate connection between PCT and infection was first discovered by French oncologist Dr. Bohuon during the Gulf War in 1991. After more than 30 years of clinical verification, the diagnostic significance of PCT has been beyond doubt. Its serum level in healthy adults is usually no more than 0.05 ng/ml, while, when exposed to bacterial infection, PCT usually rises within 2–6 h, but there is little change when exposed to viral infection (27–29). Thus, it has long been reported that PCT exhibits advantages over conventional parameters in distinguishing between bacterial and viral infections (30).

PCT elevation was associated with malignant tumor progression, which was unknown. There has been no report of PCT’s role in the prognosis of Ewing sarcoma. The previous study showed that the presence of a neuroendocrine component remained strongly associated with a positive PCT and unfavorable prognosis (31, 32). Here, we reported that the case might have a high tumor burden and poor prognosis with a high PCT level. After resection, the PCT level quickly decreased, meaning that sarcoma has a component of neuroendocrine, as Ewing sarcoma is derived from the neuroectoderm.

Moreover, we identified one and only one case of PCT high expression in another type of sarcoma, undifferentiated pleomorphic sarcoma (UPS), in the medical literature database (33). As in our case, after the exclusion of all potential sources of infection, it was speculated that the cause of the patient’s elevated PCT was most likely secondary to malignancy. This somewhat extends the availability and traceability of our case report, although PCT levels were only mildly elevated in others’ reports, and there was no decrease in PCT levels caused by surgical intervention. At present, there are few reports regarding Ewing sarcoma (or sarcoma) and PCT. More comparable cases should be collected and discussed in the future to bring new enlightenment and increase the application value of PCT.

In conclusion, the presentation of our case is intended to inspire future readers that, in addition to several tumor diseases that have been reported, in the absence of infection, high levels of PCT can be present persistently in Ewing sarcoma, although PCT has its specificity. At the same time, we highlighted the reduction of PCT due to tumor resection. This helps to broaden the significance of PCT in Ewing sarcoma. In contrast, in the absence of obvious bacterial infection, Ewing sarcoma should be included in the differential diagnosis of significantly elevated PCT, according to our experience.

## Data availability statement

The original contributions presented in the study are included in the article/supplementary materials. Further inquiries can be directed to the corresponding authors.

## Ethics statement

Written informed consent was obtained from the individual(s) for the publication of any potentially identifiable images or data included in this article.

## Author contributions

TQ guided disease treatment and contributed to the collection of original pathology and CT/MR images of the patient. YiC drafted the manuscript and contributed to the image presentation of clinical test data. MG contributed to disease surveillance and evaluation, particularly in imaging. YaC and TQ reviewed and modified the draft. All authors contributed to the article and approved the submitted version.
